# Supplementary material for: Antibiotic Acyldepsipeptides Stimulate the Streptomyces Clp-ATPase/ClpP Complex for Accelerated Proteolysis
Source: mBio. 2022 Oct 26;13(6):e01413-22. doi: 10.1128/mbio.01413-22 (PMC9765437; doi:10.1128/mbio.01413-22)
Supplement: FIG S5 [file mbio.01413-22-sf005.pdf]

SI file

Antibiotic acyldepsipeptides stimulate the *Streptomyces* Clp-ATPase/ClpP complex for accelerated proteolysis

Laura Reinhardt[1,2], Dhana Thomy[1,2], Markus Lakemeyer[3], Linda Maria Westermann[1,2], Joaquin Ortega[4], Stephan A. Sieber[3], Peter Sass[1,2,§], Heike Brötz-Oesterhelt[1,2,§,*]
[1]Department of Microbial Bioactive Compounds, Interfaculty Institute of Microbiology and Infection Medicine, University of Tübingen, Auf der Morgenstelle 28, 72076 Tübingen, Germany. [2]Cluster of Excellence - Controlling Microbes to Fight Infections, University of Tübingen, 72076 Tübingen, Germany. [3]Department of Chemistry, Technical University of Munich, Lichtenbergstraße 4, 85748 Garching, Germany. [4]Department of Anatomy and Cell Biology, McGill University, 3640 University Street, Montreal, Quebec H3A 0C7, Canada.
[*]Correspondence should be addressed to heike.broetz-oesterhelt@uni-tuebingen.de. [§]Peter Sass and Heike Brötz-Oesterhelt share senior authorship.

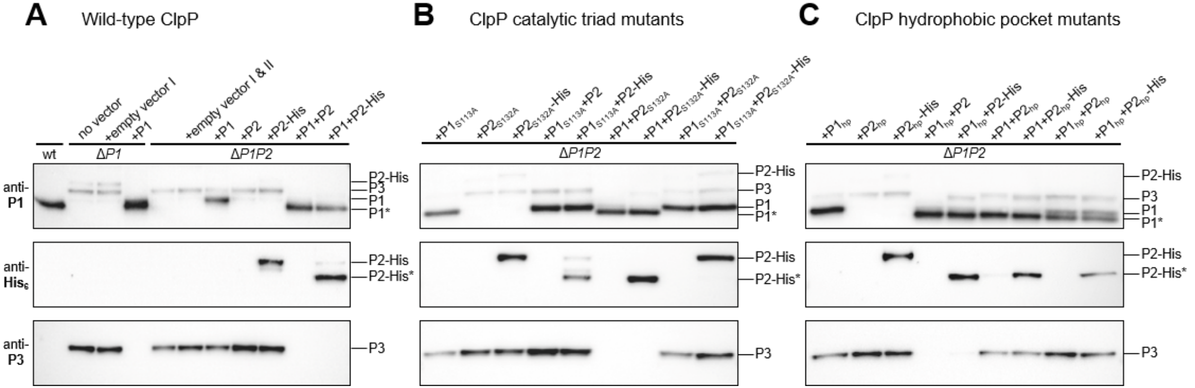

Figure S5: Cell-based studies of Clp protease activity in wild-type and *clpP1*/*clpP2* mutant cells of *S. lividans*.
