# Supplementary material for: Antibiotic Acyldepsipeptides Stimulate the Streptomyces Clp-ATPase/ClpP Complex for Accelerated Proteolysis
Source: mBio. 2022 Oct 26;13(6):e01413-22. doi: 10.1128/mbio.01413-22 (PMC9765437; doi:10.1128/mbio.01413-22)
Supplement: TABLE S1 [file mbio.01413-22-st001.pdf]

SI file

Antibiotic acyldepsipeptides stimulate the *Streptomyces* Clp-ATPase/ClpP complex for accelerated proteolysis

Laura Reinhardt[1,2], Dhana Thomy[1,2], Markus Lakemeyer[3], Linda Maria Westermann[1,2], Joaquin Ortega[4], Stephan A. Sieber[3], Peter Sass[1,2,§], Heike Brötz-Oesterhelt[1,2,§,*]
[1]Department of Microbial Bioactive Compounds, Interfaculty Institute of Microbiology and Infection Medicine, University of Tübingen, Auf der Morgenstelle 28, 72076 Tübingen, Germany. [2]Cluster of Excellence - Controlling Microbes to Fight Infections, University of Tübingen, 72076 Tübingen, Germany. [3]Department of Chemistry, Technical University of Munich, Lichtenbergstraße 4, 85748 Garching, Germany. [4]Department of Anatomy and Cell Biology, McGill University, 3640 University Street, Montreal, Quebec H3A 0C7, Canada.
[*]Correspondence should be addressed to heike.broetz-oesterhelt@uni-tuebingen.de. [§]Peter Sass and Heike Brötz-Oesterhelt share senior authorship.

Table S1. Bacterial strains and Plasmids.

Strain / plasmid		Relevant characteristic(s) / genotype	Ref. / source
E. coli			
	K-12 JM109	subcloning host	(72)
	DH5α	subcloning host	Thermo Fisher Scientific
	SG1146a	BL21(DE3) *ClpP::cam*	(78)
	ET12567 pUB307	*F-dam-13::Tn9 dcm-6 hsdM hsdR zjj-202::Tn10 recF143 galK2 galT22 ara-14 lacY1 xyl-5 leuB6 thi-1 tonA31 rpsL136 hisG4 tsx-78 mtl-1 glnV44; pUB307; Cm^R, Kan^R*	(73) (74)
S. lividans			
	TK24	*str-6;* SLP2$^-$, SLP3$^-$	(75)
	Δ*clpP1*	*str-6;* SLP2$^-$, SLP3$^-$; Δ*clpP1*	this study
	Δ*clpP1clpP2*	*str-6;* SLP2$^-$, SLP3$^-$; Δ*clpP1clpP2*	this study
Plasmids			
	pET11a	vector for the expression of native protein expression	Novagen
	pET22b	vector for the expression of C-terminal His6-fusion protein	Novagen
	pETDUET-1	vector for the co-expression of two target genes	Novagen
	pET28aShclpP1	pET28a + ORF CEB94_14110 (*S. hawaiiensis clpP1*)	this study
	pET21bShclpP2	pET21b + ORF CEB94_14105 (*S. hawaiiensis clpP2*)	this study
	pET11aShclpP1$_{ATG2}$	pET11a + ORF CEB94_14110 (*S. hawaiiensis clpP1*)	this study
	pET11aShclpP2	pET11a + ORF CEB94_14105 (*S. hawaiiensis clpP2*)	this study
	pET22bShclpP1$_{ATG2}$-His6	pET22b + ORF CEB94_14110 (*S. hawaiiensis clpP1*)	this study
	pET22b**Nco*I-ShclpP2-His6	pET22b**Nco*I + ORF CEB94_14105 (*S. hawaiiensis clpP2*)	this study
	pET22b**Nco*I-ShclpP2$_{ATG2}$-His6	pET22b**Nco*I + ORF CEB94_14105 (*S. hawaiiensis clpP2*)	this study
	pET22bShclpP1*-His6	pET22b + ORF CEB94_14110 (*S. hawaiiensis clpP1*)	this study
	pET22b**Nco*I-ShclpP2*-His6	pET22b + ORF CEB94_14105 (*S. hawaiiensis clpP2*)	this study
	pET11aShclgR-N-His6	pET11a + ORF CEB94_30145 (*S. hawaiiensis clgR*)	this study
	pET11aShpopR-N-His-6	pET11a + MT943519 (*S. hawaiiensis popR*)	this study
	pETDUETShclpP1$_{ATG2}$clpP2-His6	pETDUET-1 + ORF CEB94_14110 + 14105 (*S. hawaiiensis clpP1+P2*)	this study
	pET22b**Nco*I-ShclpX-His6	pET22b**Nco*I + ORF CEB94_14100 (*S. hawaiiensis clpX*)	this study
	pET22b**Nco*I-ShclpC1-His6	pET22b*NcoI + ORF CEB94_23085 (*S. hawaiiensis clpC1*)	this study
	pET22bShclpC2-His6	pET22b + ORF CEB94_33910 (*S. hawaiiensis clpC2*)	this study
	pET22bShclpP1$_{S113A}$	pET22bshclpP1$_{ATG2}$-His6 carrying aa mutation S113A in the *S. hawaiiensis clpP1* gene	this study

pET22bShclpP2$_{S131A}$	pET22b**Nco*I-ShclpP2-His6 carrying aa mutation S131A in the *S. hawaiiensis clpP2* gene	this study
pET11aShclpP1$_{hp}$	pET11aShclpP1$_{ATG2}$ carrying aa mutation Y76V, Y78V, Y98V in the *S. hawaiiensis clpP1* gene	this study
pET22bShclpP2$_{hp}$	pET22b**Nco*I-ShclpP2$_{ATG2}$-His6 carrying aa mutation S94A, Y96V, Y116V in the *S. hawaiiensis clpP2* gene	this study
pET11aShclpP1$_{Y76SATG2}$	pET11aShclpP1$_{ATG2}$ carrying aa mutation Y76S in the *S. hawaiiensis clpP1* gene	this study
pET22b**Nco*I-ShclpP2$_{S94YATG2}$-His6	pET22b**Nco*I-ShclpP2$_{ATG2}$-His6 carrying aa mutation S94Y in the *S. hawaiiensis clpP2* gene	this study
pGM-GUS	temperature-sensitive *Streptomyces* shuttle vector *aac(3)IV, oriT,* P_{ermE}*_gusA, rep*$_{ts}$	Günther Muth, Tübingen
pGM-GUS-Xba	based on pGM-GUS, introduction of an XbaI restriction site by site-directed mutagenesis	this study
pGM-GUS-clpP1	knockout vector for *S. lividans clpP1*	this study
pGM-GUS-clpP1clpP2	knockout vector for *S. lividans clpP2clpP2*	this study
pIJ12551	ΦC31-integrative Streptomyces shuttle vector, protein expression under *ermE** promotor	(76)
pIJ12551clpP1	constitutive protein expression of SlClpP1	this study
pIJ12551clpP1$_{S113A}$	constitutive protein expression of SlClpP1 with the following mutation(s): S113A	this study
pIJ12551clpP1$_{hp}$	constitutive protein expression of SlClpP1 with the following mutation(s): Y76V, Y78V, Y98V	this study
pIJ12551clpP1clpP2	constitutive protein expression of SlClpP1ClpP2	this study
pIJ10257	ΦBT1-integrative Streptomyces shuttle vector, protein expression under *ermE** promotor	(77)
pIJ10257clpP2	constitutive expression of SlClpP2	this study
pIJ10257clpP2-His	constitutive expression of SlClpP2 with an N-terminal 6xHis-tag	this study
pIJ10257clpP2$_{S132A}$	constitutive protein expression of SlClpP2 with the following mutation(s): S132A	this study
pIJ10257clpP2-His$_{S132A}$	constitutive protein expression of SlClpP2 with an N-terminal 6xHis-tag with the following mutation(s): S132A	this study
pIJ10257clpP2$_{hp}$	constitutive protein expression of SlClpP2 with the following mutation(s): S95A, Y97V, Y117V	this study
pIJ10257clpP2-His$_{hp}$	constitutive protein expression of SlClpP2 with an N-terminal 6xHis-tag with the following mutation(s): S95A, Y97V, Y117V	this study
