# Supplementary material for: Antibiotic Acyldepsipeptides Stimulate the Streptomyces Clp-ATPase/ClpP Complex for Accelerated Proteolysis
Source: mBio. 2022 Oct 26;13(6):e01413-22. doi: 10.1128/mbio.01413-22 (PMC9765437; doi:10.1128/mbio.01413-22)
Supplement: TABLE S2 [file mbio.01413-22-st002.pdf]

SI file

Antibiotic acyldepsipeptides stimulate the *Streptomyces* Clp-ATPase/ClpP complex for accelerated proteolysis

Laura Reinhardt[1,2], Dhana Thomy[1,2], Markus Lakemeyer[3], Linda Maria Westermann[1,2], Joaquin Ortega[4], Stephan A. Sieber[3], Peter Sass[1,2,§], Heike Brötz-Oesterhelt[1,2,§,*]
[1]Department of Microbial Bioactive Compounds, Interfaculty Institute of Microbiology and Infection Medicine, University of Tübingen, Auf der Morgenstelle 28, 72076 Tübingen, Germany. [2]Cluster of Excellence - Controlling Microbes to Fight Infections, University of Tübingen, 72076 Tübingen, Germany. [3]Department of Chemistry, Technical University of Munich, Lichtenbergstraße 4, 85748 Garching, Germany. [4]Department of Anatomy and Cell Biology, McGill University, 3640 University Street, Montreal, Quebec H3A 0C7, Canada.
[*]Correspondence should be addressed to heike.broetz-oesterhelt@uni-tuebingen.de. [§]Peter Sass and Heike Brötz-Oesterhelt share senior authorship.

Table S2. Primer. Restriction sites are underlined.

Plasmid		Forward (F)/Reverse (R) oligo (5'- 3' direction)	Template
pET11aShclpP1$_{ATG2}$		F: aaacatatgacgaatctgatgccctcagc	pET28aShclpP1
		R: aaaggatcctcaggccccggtgccgc	
pET11aShclpP2		F: aaacatatgaaccagttccccggcag	*S. hawaiiensis* genomic DNA
		R: aaaggatcctcagcgcaggctcgagttgtc	
pET22bShclpP1$_{ATG2}$-His6		F: aaacatatgacgaatctgatgccctcagc	pET28aShclpP1
		R: taaactcgagggccccggtgccgcc	
pET22b**Nco*I-ShclpP2-His6		F: aaacatatgaaccagttccccggcag	*S. hawaiiensis* genomic DNA
		R: aaaccatgggcgcaggctcgagttgtc	
pET22b**Nco*I-ShclpP2$_{ATG2}$-His6		F: aaacatatgagcgcctcccagggc	pET21bShclpP2
		R: aaaccatgggcgcaggctcgagttgtc	
pET22bShclpP1*-His6		F: aaacatatgccttccatcggtggtggcctcggt	pET22bShclpP1$_{ATG2}$-His6
		R: taaactcgagggccccggtgccgcc	
pET22b**Nco*I-ShclpP2*-His6		F: aaacatatgcgctacatcattccccgcttc	pET22b**Nco*I-ShclpP2-His6
		R: aaaccatgggcgcaggctcgagttgtc	
pET11aShclgR-N-His6		F: aaacatatgcaccaccaccaccaccacattctgctccgtcgcctgctgggtgacgtg	*S. hawaiiensis* genomic DNA
		R: taaggatcctcacgcggcgacgacgtccactgc	
pET11aShpopR-N-His-6		F: aaacatatgcaccaccac caccaccacaccagccacctgccgaacgaagcccgagtc	*S. hawaiiensis* genomic DNA
		R: taaggatcctcaggcggccaggcacattccgtcgtg	
pETDUETShclpP1$_{ATG2}$clpP2-His6	clpP1	F: aaacatatgacgaatctgatgccctcagc	pET11aShclpP1$_{ATG2}$
		R: aaaggtacctcaggccccggtgcc	
	clpP2	F: aaaccatgggaaaccagttccccggcagcg	pET22b**Nco*I-ShclpP2-His6
		R: aaaaagctttcagtggtggtggtggtggtggcgcaggctcgagttgtccatcttcc	
pET22b**Nco*I-ShclpX-His6		F: aaacatatggcacgcatcggtgacggcg	genomic DNA *S. hawaiiensis*
		R: aaaccatggggccgtcttctgctcccccgg	
pET22b**Nco*I-ShclpC1-His6		F: aagcatatgttcgagaggttcaccgacc	genomic DNA *S. hawaiiensis*
		R: aaaccatggggcgtccttgctcaggttcggg	
pET22bShclpC2-His6		F: aaacatatgagcagcggcttcaccagc	*S. hawaiiensis* genomic DNA
		R: t aaactcgagtcggggcacggtactgaacg	
pET22bShclpP1$_{S113A}$		F: gggcctggcagccgcgatgggccagttc	pET22bShclpP1$_{ATG2}$-His6
		R: gaactggcccatcgcggctgccaggccc	
pET22bShclpP2$_{S131A}$		F: ccaggcggccgccgccgccgccgtc	pET22b**Nco*I-ShclpP2-His6
		R: gacggcggcggcggcggccgcctgg	

pET11aShclpP1$_{hp}$	F: ggagaaggacatcgtcctggtcatcaacagccccggc	pET11aShclpP1$_{ATG2}$
	R: cctcttcctgtagcaggaccagtagttgtcggggccg	
	F: gacaccatgcaggtcatcaagaacgac	
	R: gtcgttcttgatgacctgcatggtgtc	
pET22bShclpP2$_{hp}$	F: cgaccgtgacatcgcggtggtcatcaacagccccggc	pET22b**Nco*I-ShclpP2$_{ATG2}$-His6
	R: gctggcactgtagcgccaccagtagttgtcggggccg	
	F: gacacgatgcaggtcgtgaagccggac	
	R: gtccggcttcacgacctgcatcgtgtc	
pET11aShclpP1$_{Y76S}$	F: gagaaggacatctccctgtacatcaacag	pET11aShclpP1$_{ATG2}$
	R: ctgttgatgtacagggagatgtccttctc	
pET22bShclpP2$_{S94YATG2}$-His6	F: gaccgtgacatctacgtgtacatcaac	pET22b**Nco*I-ShclpP2$_{ATG2}$-His6
	R: gttgatgtacacgtagatgtcacggtc	
pGM-GUS-Xba	F: cccgcgcccagtccgagctctagacggcgcttttcacctggc	pGM-GUS
	R: gccaggtgaaaagcgccgtctagagctcggactgggcgcggg	
pGM-GUS-clpP1	F1: ctgcagacgcgtcgacgtcatatgacatcacggagctgaag	pGM-GUS-Xba,
	R1: cgatcgtctgcacgtatccacctgctcg	*S. lividans* TK24 genomic DNA
	F2: tggatacgtgcagacgatcgagcagatc	
	R2: ccacggcgatatcggatccatatgccagcaggaggatgttgg	
pGM-GUS-clpP1clpP2	F1: tggctccaattgtacatcggatccatatggacatcacggagctgaag	pGM-GUS-Xba,
	R1: tggtgatgatctggtccacgtatccacctgctcg	*S. lividans* TK24 genomic DNA
	F2: aggtggatacgtggaccagatcatcaccaccc	
	R2: agcttctgcagacgcgtcgacgtcatatggaatcccgggggatcagc	
pIJ12551clpP1	F: ggaattccatatgacgaatctgatgccctcag	pIJ12551,
	R: atagtttagcggccgctcaggcgcccgtgccgccgc	*S. lividans* TK24 genomic DNA
pIJ12551clpP1$_{S113A}$	F: gcgatgggtctcgcggccGccatgggacagttcctgc	pIJ12551clpP1
	R: gcaggaactgtcccatggcggccgcgagacccatcgc	
pIJ12551clpP1$_{hp}$	F1: gacccggacaaggacatcGTcctgGTcatcaacagcccgggcgg	pIJ12551clpP1
	R1: ccgcccgggctgttgatgaccaggacgatgtccttgtccgggtc	
	F2: gatctacgacaccatgcagGTcatcaagaacgacgtggtg	
	R2: caccacgtcgttcttgatgacctgcatggtgtcgtagatc	
pIJ12551clpP1clpP2	F: ggaattccatatgacgaatctgatgccctcag	pIJ12551,
	R: atagtttagcggccgcctagcggagagaggagttgtc	*S. lividans* TK24 genomic DNA
pIJ10257clpP2	F: ggaattccatatgaacgacttccccggcagcg	pIJ10257,
	R: cccaagcttctagcggagagaggagttgtc	*S. lividans* TK24 genomic DNA
pIJ10257clpP2-His	F: ggaattccatatgaacgacttccccggcagcg	pIJ10257,
	R: cccaagcttctaatgatgatgatgatgatggcggagagaggagttgtc	*S. lividans* TK24 genomic DNA

pIJ10257clpP2$_{S131A}$	F: gtctgcatgggccaggccgccgccgccgccgccgtcctgctgg	pIJ10257clpP2
	R: ccagcaggacggcggcggcggcggcggcctggcccatgcagac	
pIJ10257clpP2-His$_{S132A}$	F: gtctgcatgggccaggccgccgccgccgccgccgtcctgctgg	pIJ10257clpP2-His
	R: ccagcaggacggcggcggcggcggcggcctggcccatgcagac	
pIJ10257clpP2$_{hp}$	F1: cccgaccgggacatcgcggtcgtcatcaacagccccgg	pIJ12057clpP2
	R1: ccggggctgttgatgacgaccgcgatgtcccggtcggg	
	F2: ctacgacacgatgcaggtcgtgaagccggacgtccagac	
	R2: gtctggacgtccggcttcacgacctgcatcgtgtcgtag	
pIJ10257clpP2-His$_{hp}$	F1: cccgaccgggacatcgcggtcgtcatcaacagccccgg	pIJ12057clpP2-His
	R1: ccggggctgttgatgacgaccgcgatgtcccggtcggg	
	F2: ctacgacacgatgcaggtcgtgaagccggacgtccagac	
	R2: gtctggacgtccggcttcacgacctgcatcgtgtcgtag	
